# Endovascular repair of aortic coarctation and associated aneurysms with a thoracic branched endoprosthesis

**DOI:** 10.1016/j.xjse.2024.100018

**Published:** 2024-08-22

**Authors:** Jonathan P. Putnam, Matthew A. Thompson, Benjamin P. Kramer, Francis J. Caputo, Margaret Fuchs, Alberto J. Lopez, Xiaoying Lou, Juan P. Umana, Eric E. Roselli

**Affiliations:** a Department of Thoracic and Cardiovascular Surgery, Heart, Vascular, and Thoracic Institute, Cleveland Clinic, Cleveland, Ohio; b The Aorta Center, Heart, Vascular, and Thoracic Institute, Cleveland Clinic, Cleveland, Ohio; c Department of Vascular Surgery, Heart, Vascular, and Thoracic Institute, Cleveland Clinic, Cleveland, Ohio; d Department of Cardiovascular Medicine, Heart, Vascular, and Thoracic Institute, Cleveland Clinic, Cleveland, Ohio; e The Adult Congenital Cardiac Center, Heart, Vascular, and Thoracic Institute, Cleveland Clinic, Cleveland, Ohio; f Department of Vascular Surgery, Cleveland Clinic Florida, Weston, Fla; g Department of Thoracic and Cardiovascular Surgery, Cleveland Clinic Florida, Weston, Fla

**Figure:**
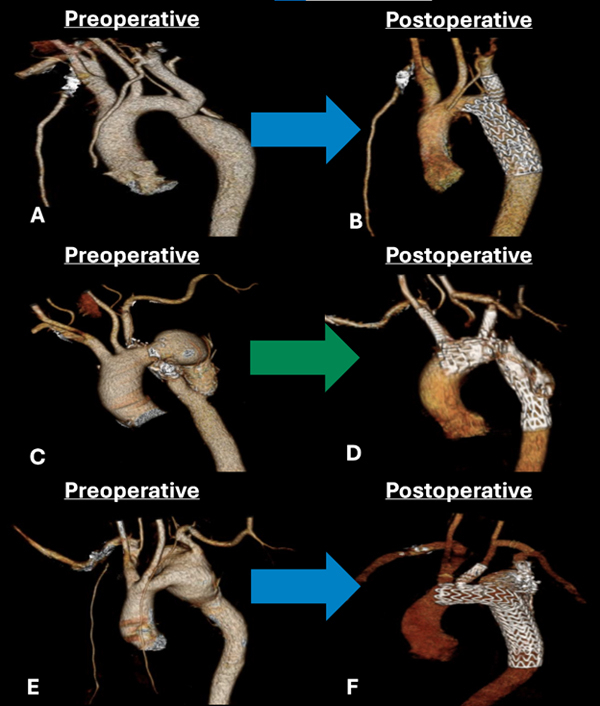
A and B, Primary CoA. C through F, Post-CoA pseudoaneurysm/aneurysm repaired with TBE.

**Central Message**

The thoracic branched endoprosthesis enables complete endovascular repair of aortic coarctation and associated aneurysms in both primary and complex reoperative scenarios in adults.

Endovascular stent grafts provide durable repair of aortic coarctation and coarctation-associated aneurysms in adults.^1^^,^^2^ The GORE TAG thoracic branched endoprosthesis (TBE) (W. L. Gore & Associates Inc), which consists of a main aortic stent graft and a side branch component, facilitates endovascular repair in appropriately selected patients. We present 3 cases of coarctation repair using a TBE, demonstrating its versatility to address both adult congenital and acquired aortic disease in primary and complex reoperative settings. Patient consent was obtained and documented in accordance with institutional review board guidelines; approval not required.

## Case 1

A 21-year-old woman presented with hypertension taking 4 antihypertensive medications. Computed tomography (CT) showed a 9 × 6 mm coarctation at the aortic isthmus, a dilated left subclavian artery, and a nondominant left vertebral artery originating from aortic zone 2 (Video 1). Mean transcoarct gradient was 30 mm Hg by direct measurement. The patient underwent elective endovascular repair with a TBE. Percutaneous access was obtained via right common femoral and left brachial arteries. A 26 × 100 mm main aortic device was deployed from zone 2 to zone 4. The left subclavian artery was bridged with a 15 × 60 mm side branch device. The device was expanded within the coarctation to full diameter and there was no residual gradient. The patient was discharged on postoperative day 3. Follow-up CT at 3 months showed a patent endograft and patent arch branch vessels without recoarctation or endoleak. She was then weaned to 2 antihypertension medications.

## Case 2

**Figure 1:**
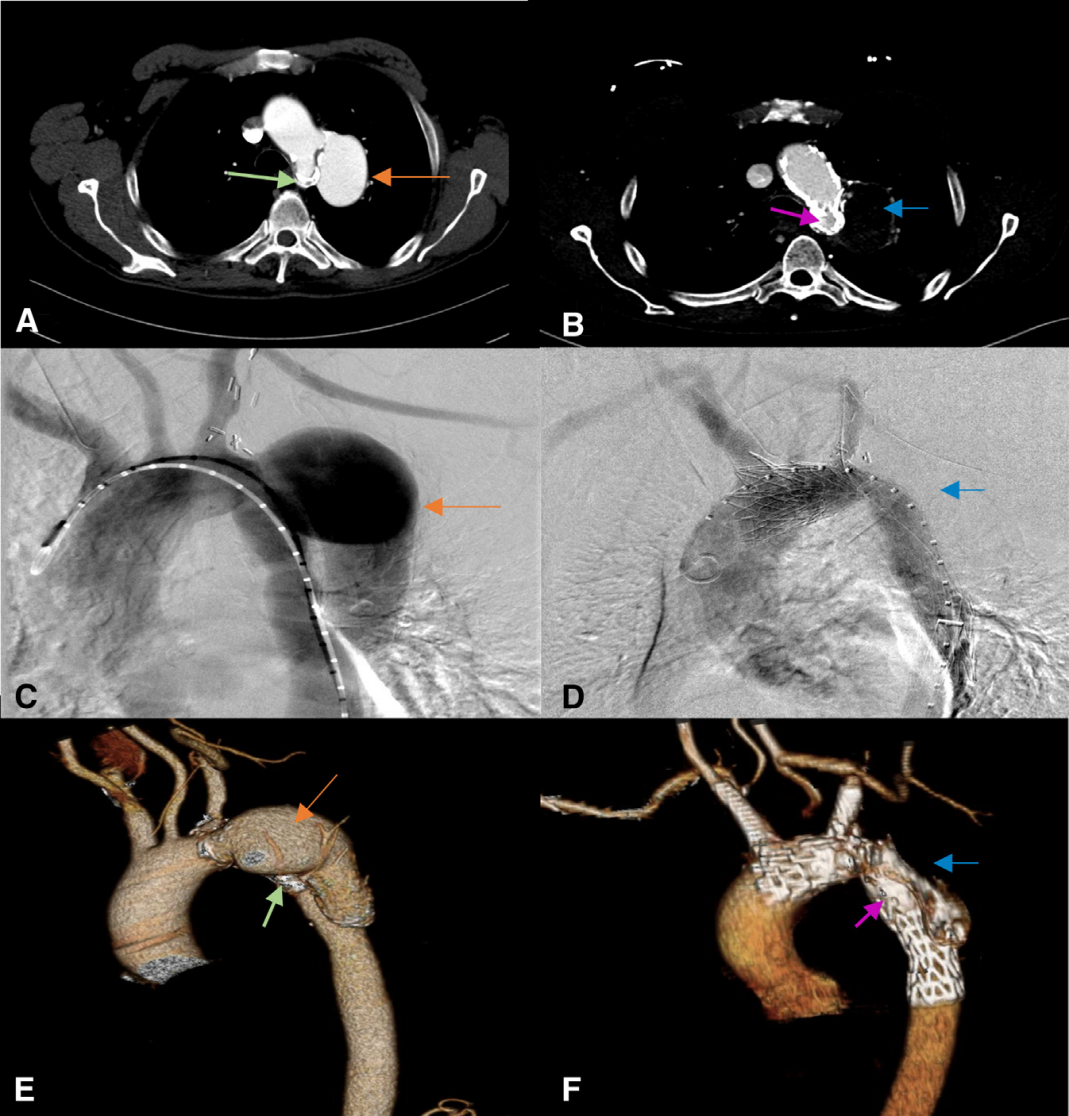
Preoperative computed tomography (*CT*) (A), digital subtraction angiography (*DSA*) (C), and 3-dimensional reconstruction (E) demonstrate pseudoaneurysm of the extra-anatomic bypass (*orange arrow*) implanted to address anastomotic coarctation of in situ graft (*green arrow*). Postoperative CT (B), DSA (D), and 3-dimensional reconstruction (F) demonstrate successful stent dilation of recoiled interposition graft (*purple arrow*) and exclusion of the extra-anatomic bypass graft (*blue arrow*) using a thoracic branched endoprosthesis device.

A 69-year-old man with a history of traumatic descending thoracic aortic rupture 50 years prior, repaired with an interposition graft, complicated by an anastomotic coarctation repaired with arch-to-zone 4 extra-anatomic bypass, presented with a 4.9-cm pseudoaneurysm of the proximal aspect of the bypass graft (Figure 1, *A*, *C*, and *E* and Video 1). Given his complex anatomy, he underwent a hybrid repair comprising first-stage left subclavian-left common carotid bypass and second stage TBE repair with coverage extending across the left common carotid artery. Percutaneous access was performed as above. A 34 × 150 mm main aortic device was deployed from zone 2 to zone 4 to re-expand the anastomotic coarctation. The left subclavian artery was bridged with a 10 × 60 mm side branch device. Angiography revealed persistent flow into the aneurysmal bypass graft. A 37 × 40 mm proximal extension cuff was deployed in zone 1 to fully exclude the aneurysm and cover the left common carotid artery. Because of the proximity of the extension cuff to the innominate artery orifice, a 9 × 60 mm Express LD bare metal stent (Boston Scientific) was deployed in the innominate and right common carotid arteries. The patient was discharged on postoperative day 4. Follow up CT at 3 months showed thrombosis of the aneurysmal bypass graft (Figure 1, *B*, *D*, and *F*).

## Case 3

**Figure 2:**
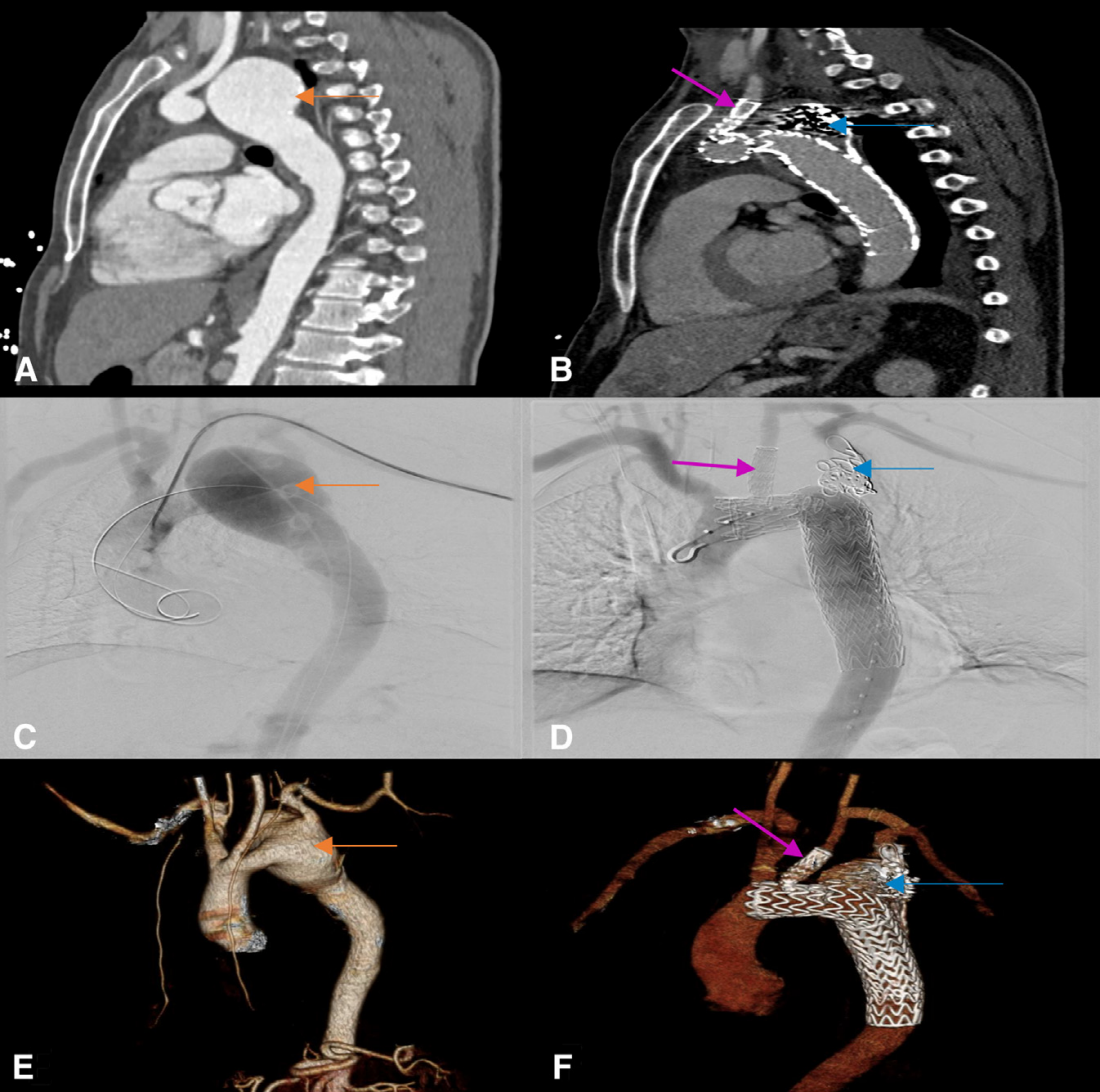
Preoperative computed tomography (*CT*) (A), digital subtraction angiography (*DSA*) (C), and 3-dimensional reconstruction (E) demonstrate aneurysmal dilation of the proximal descending aorta (*orange arrow*) involving the takeoff of the left subclavian artery. Postoperative CT (B), DSA (D), and 3-dimensional reconstruction (F) demonstrate thoracic branched endoprosthesis side branch placement into the left common carotid artery (*purple arrow*) with extensive coiling of the left subclavian artery (*blue arrow*).

A 43-year-old man with a history of coarctation repair at age 15 months presented with acute dysphagia. CT demonstrated tracheal and esophageal displacement by a 6.0 cm proximal descending thoracic aorta aneurysm involving the left subclavian artery (Figure 2, *A*, *C*, and *E*). His morphology necessitated zone 1 repair through a hybrid approach comprising first-stage left common carotid-left subclavian bypass and second-stage TBE repair. Percutaneous access was performed as above but with the left brachial guidewire traversing the carotid to subclavian bypass and entering the aorta through the left common carotid artery. A 26 × 100 mm stent graft was deployed in the descending thoracic aorta to exclude the distal portion of the aneurysm. Then, a 26 × 150 mm TBE main aortic device was deployed from zone 1 to zone 4. The left common carotid artery was bridged with a 10 × 6 mm side branch device. Coil embolization of the left subclavian artery beyond the aneurysm was performed to prevent type II endoleak. The patient was discharged on postoperative day 2 with symptomatic relief. Follow-up CT at 1 week showed aneurysmal exclusion (Figure 2, *B*, *D*, and *F*).

## Conclusions

The prevalence of adult coarctation is rising with advancements in detection and management.^1^^,^^3^ Some long-term survivors of coarctation repair are at risk for late complications like aneurysm, pseudoaneurysm, and recurrent coarctation. Novel therapies and devices expand options for surgeons treating this growing population. Transcatheter solutions are ideal for isolated lesions without concomitant cardiovascular pathology given the tenets of endovascular repair are upheld. Aggressive balloon molding with compliant (eg, Balloon Catheter; Coda) or noncompliant (eg, True Dilatation Balloon Valvuloplasty Catheter; BD) options is often needed to expand the coarcted segment. A proximal landing zone diameter of 16 mm in women and 18 mm in men is crucial for complete seal and unimpeded flow. The TBE offers the advantages of endovascular repair while maintaining aortic branch vessel perfusion and additional versatility in patients with more complex arch branch vessel anatomy.^4^^,^^5^ The TBE's design reduces the need for debranching procedures and redo thoracotomy while remaining effective in hybrid palliation. Aortic surgeons should be aware of TBE devices as a valuable tool for treating coarctation and late coarctation-associated complications in adults.

## Conflict of Interest Statement

Dr Roselli has relationships with Artivion, Cook, Edwards Lifesciences, Medtronic, Terumo Aortic, and W.L. Gore as a speaker, consultant, and investigator. All other authors reported no conflicts of interest.

The *Journal* policy requires editors and reviewers to disclose conflicts of interest and to decline handling or reviewing manuscripts for which they may have a conflict of interest. The editors and reviewers of this article have no conflicts of interest.

## Supplementary Data

Video 1 Operative video of Case 1 and Case 2 as presented at The American Association for Thoracic Surgery Aortic Symposium 2024, New York, New York, April 25-26, 2024. Video available at: https://www.jtcvs.org/article/S2950-6050(24)00018-4/fulltext.
