# Supplementary figures and images for: Endovascular repair of aortic coarctation and associated aneurysms with a thoracic branched endoprosthesis
Source: JTCVS Struct Endovasc. 2024 Aug 22;3:100018. doi: 10.1016/j.xjse.2024.100018 (PMC13244734; doi:10.1016/j.xjse.2024.100018)

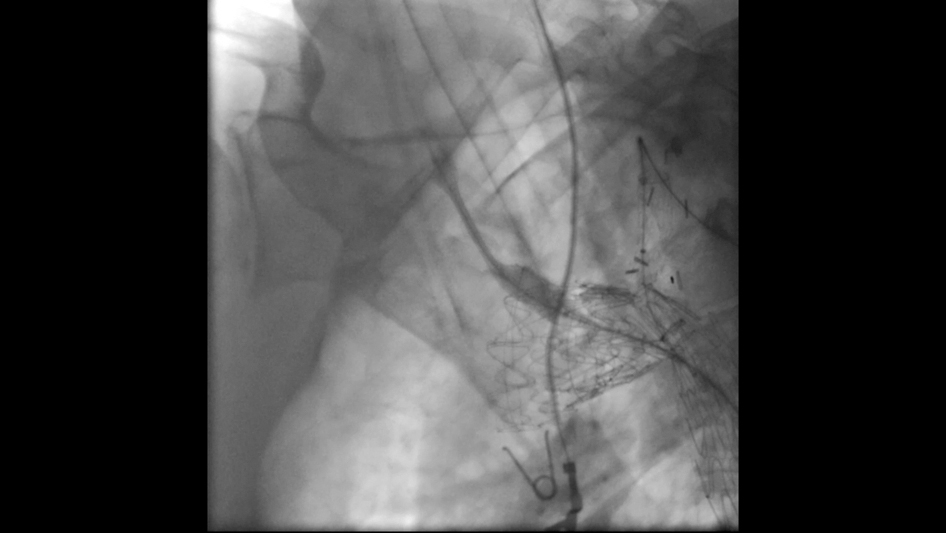

Supplement: Video 1 — Operative video of Case 1 and Case 2 as presented at The American Association for Thoracic Surgery Aortic Symposium 2024, New York, New York, April 25-26, 2024. Video available at: https://www.jtcvs.org/article/S2950-6050(24)00018-4/fulltext. [file fx2.jpg]
